# COVID-19 related outcomes among individuals with neurodegenerative diseases: a cohort analysis in the UK biobank

**DOI:** 10.1186/s12883-021-02536-7

**Published:** 2022-01-07

**Authors:** Yihan Hu, Huazhen Yang, Can Hou, Wenwen Chen, Hanyue Zhang, Zhiye Ying, Yao Hu, Yajing Sun, Yuanyuan Qu, Maria Feychting, Unnur Valdimarsdottir, Huan Song, Fang Fang

**Affiliations:** 1 West China Biomedical Big Data Center and National Clinical Research Center for Geriatrics, West China Hospital, Sichuan University, Guo Xue Lane 37, Chengdu, 610041 China; 2 Medical Big Data Center, Sichuan University, Chengdu, 610041 China; 3 Institute of Environmental Medicine, Karolinska Institutet, 171 77 Stockholm, Sweden; 4 Division of Nephrology, Kidney Research Institute, State Key Laboratory of Biotherapy and Cancer Center, West China Hospital, Sichuan University, Chengdu, 610041 China; 5 Center of Public Health Sciences, Faculty of Medicine, University of Iceland, 101 Reykjavík, Iceland; 6 Department of Medical Epidemiology and Biostatistics, Karolinska Institutet, 171 77 Stockholm, Sweden

**Keywords:** Neurodegenerative diseases, COVID-19, Cohort study, UK biobank

## Abstract

**Background:**

An increased susceptibility to COVID-19 has been suggested for individuals with neurodegenerative diseases, but data are scarce from longitudinal studies.

**Methods:**

In this community-based cohort study, we included 96,275 participants of the UK Biobank who had available SARS-CoV-2 test results in Public Health England. Of these, 2617 had a clinical diagnosis of neurodegenerative diseases in the UK Biobank inpatient hospital data before the outbreak of COVID-19 (defined as January 31st, 2020), while the remaining participants constituted the reference group. We then followed both groups from January 31st, 2020 to June 14th, 2021 for ascertainment of COVID-19 outcomes, including any COVID-19, inpatient care for COVID-19, and COVID-19 related death. Logistic regression was applied to estimate the association between neurogenerative disease and risks of COVID-19 outcomes, adjusted for multiple confounders and somatic comorbidities.

**Results:**

We observed an elevated risk of COVID-19 outcomes among individuals with a neurodegenerative disease compared with the reference group, corresponding to a fully adjusted odds ratio of 2.47 (95%CI 2.25–2.71) for any COVID-19, 2.18 (95%CI 1.94–2.45) for inpatient COVID-19, and 3.67 (95%CI 3.11–4.34) for COVID-19 related death. Among individuals with a positive test result for SARS-CoV-2, individuals with neurodegenerative diseases had also a higher risk of COVID-19 related death than others (fully adjusted odds ratio 2.08; 95%CI 1.71–2.53).

**Conclusion:**

Among UK Biobank participants who received at least one test for SARS-CoV-2, a pre-existing diagnosis of neurodegenerative disease was associated with a subsequently increased risk of COVID-19, especially COVID-19 related death.

**Supplementary Information:**

The online version contains supplementary material available at 10.1186/s12883-021-02536-7.

## Background

The coronavirus disease 2019 (COVID-19), caused by severe acute respiratory syndrome coronavirus 2 (SARS-CoV-2), was declared to be a pandemic by World Health Organization (WHO) in March 2020 [1]. As of September 28th 2021, the disease had led to 232,075,351 confirmed cases and 4,752,988 cases of death. Although most individuals contracted with COVID-19 experience rather mild symptoms [2], elderly people and individuals with chronic diseases are more prone to experience a severe disease course [3–6].

Neurodegenerative diseases are a group of diseases with progressive loss of neurons in the central nervous system. Alzheimer’s disease (AD) is the most common neurodegenerative disease, characterized by impairment in memory, language, and problems in daily living activities, and is also commonly accompanied with non-cognitive symptoms such as depression [7]. AD affects about 5–7% of the population aged over 65 years worldwide [8]. With the early death of dopaminergic neurons, Parkinson’s disease (PD) mainly affects the motor system, leading to movement disorders typically presented as rest tremor, rigidity, bradykinesia, postural, and gait impairment [9]. Individuals with neurodegenerative diseases are a high-risk population during the COVID-19 pandemic, because of their advanced age and presence of other comorbidities [10–12]. Self-isolation and social distancing due to COVID-19 may have further influence on individuals living with neurodegenerative diseases [13].

To date, only a handful of studies have assessed the association between neurodegenerative diseases and COVID-19, with inconclusive findings [14–22]. This inconsistency may be attributable to different study design, varying sample size and the resulting statistical power, and varying degree of control for important confounders (e.g., comorbidity, lifestyle, and socioeconomic status). We summarized findings of the existing studies in Supplementary Table 1.

To this end, taking advantage of the rich information on lifestyle factors and the complete and timely updated follow-up data for somatic disease and COVID-19 infection in the UK Biobank, we aimed to assess the association between previous neurodegenerative diseases, including primary and vascular neurodegenerative diseases, and subsequent risk of COVID-19.

## Methods

### Study design

UK Biobank is a large prospective study that recruited 502,507 participants aged 40–69 years between 2006 and 2010. Baseline data such as demographical factors, lifestyle, and socioeconomic status were collected at recruitment. To ascertain health-related outcomes, the cohort is periodically linked to multiple national datasets [23]. UK Biobank inpatient hospital data include data extracted from Hospital Episode Statistics (HES) from England, Patient Episode Database for Wales (PEDW) from Wales, and the Scottish Morbidity Record from Scotland. Mortality data are derived from multiple death registers, including NHS digital in England and Wales, and NHS central register in Scotland. After the start of COVID-19 outbreak in the UK, COVID-19 test results from Public Health England (PHE), based on real-time RT-PCR (RdRp gene assay) for nose and throat swabs, have also been linked to UK Biobank with updates on a monthly basis.

**Fig. 1:**
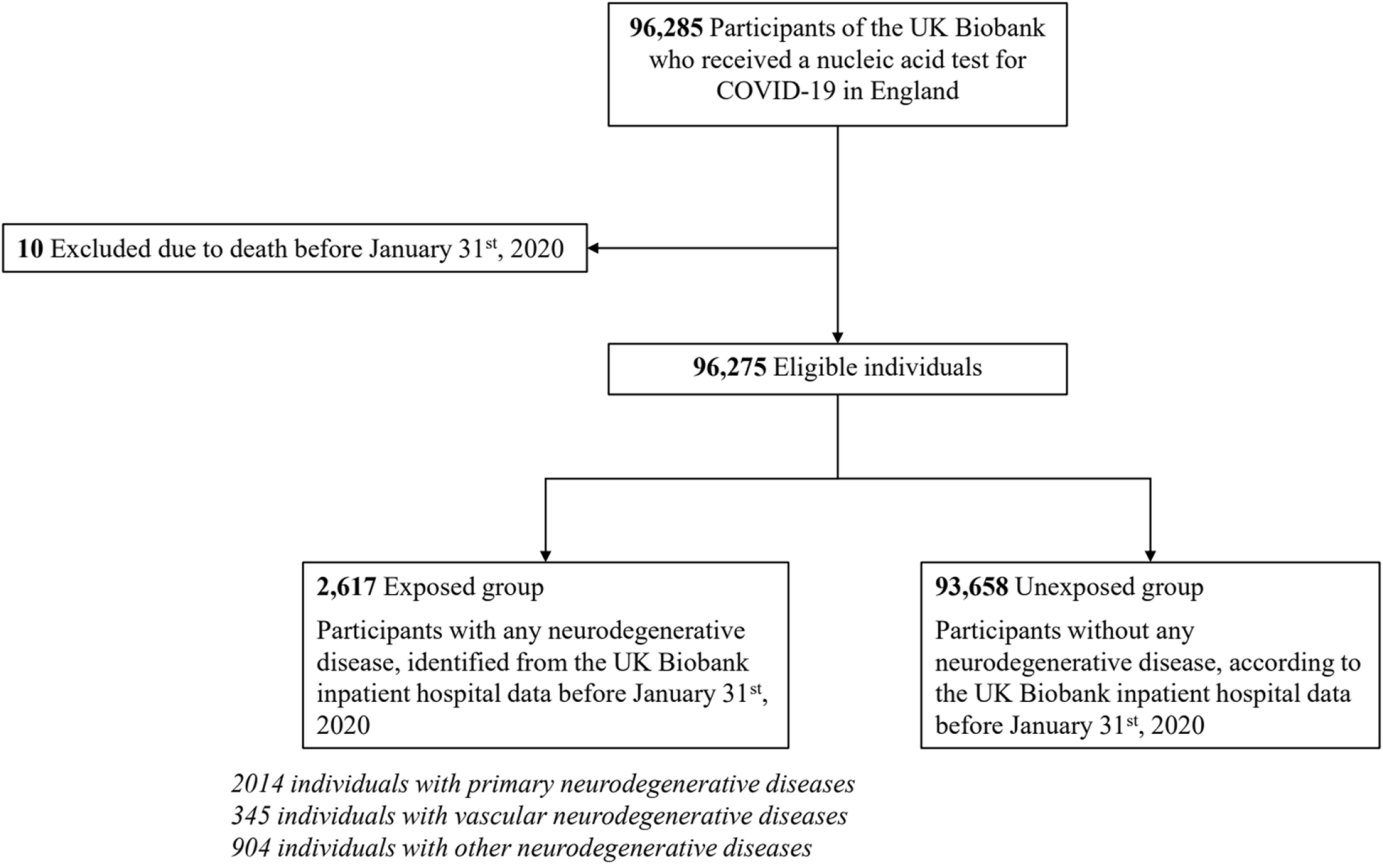
Study design. ^a^ The first case of COVID-19 was diagnosed on January 31st 2020 in the UK

To reduce the possible bias in COVID-19 detection due to testing capacity, especially in the beginning of the pandemic, in the UK [24], we restricted our analysis to UK Biobank participants who had at least one COVID-19 test record from PHE before June 14th, 2021 (*n* = 96,285). We further excluded ten participants who were found out to have died before January 31st 2020, leaving 96,275 individuals in the final analyses (Fig. 1). Individuals with an inpatient admission record of any neurodegenerative disease before COVID-19 outbreak (i.e., January 31st, 2020, when the first COVID-19 case was diagnosed in the UK) were included in the exposed group, with the remaining participants included in the unexposed group.

Participation in UK Biobank was voluntary, and all participants gave informed consent before data collection. Ethical approval was received from NHS Research Ethics Committee (REC reference: 16/NW/0274). The present study was approved by the West China Hospital (reference number: 2020.661).

#### Neurodegenerative diseases

We identified individuals with neurodegenerative diseases on the basis of UK Biobank inpatient hopital data, according to International Classification of Diseases codes (ICD-10 and ICD-9). The definition of any neurodegenerative disease was a combinition of primary neurodegenerative diseases (ICD-10: F00, F02.0, F02.3, G12.2, G20, G23.1, G23.2, G23.8, G23.9, G25.9, G30, G31.0, G31.1, G31.8, and G31.9, ICD-9: 290.0, 290.1, 331.0, 331.1, 331.2, 331.9, 332.0, 333.0, and 335.2), vascular neurodegenerative diseases (ICD-10: F01, and G21.4, ICD-9: 290.4), and other neurodegenerative diseases (ICD-10: F03, and F05.1, ICD-9: 290.8, and 290.9). In sub-analyses, we also sepcifically analyzed AD (ICD-10: F00, G30, ICD-9: 290.0, 290.1, 331.0) and PD (ICD-10: G20, ICD-9: 332.0). All ICD codes are listed in Supplementary Table 2.

#### Ascertainment of COVID-19

By severity, we classified the outcomes of interest as any COVID-19 diagnosis, inpatient COVID-19, and COVID-19 related death. Any COVID-19 was defined in case of a positive test result for COVID-19 from PHE, an inpatient diagnosis of COVID-19 from UK Biobank inpatient hospital data, or a death with a cause concerning COVID-19 in the death registers. Inpatient COVID-19 referred to a positive test result from PHE with the origin of inpatient care, or an inpatient diagnosis of COVID-19 based on UK Biobank inpatient hospital data. COVID-19 related death was a death with a cause of death concerning COVID-19 in the death registers. We used ICD-10 codes U07.1 or U07.2 to ascertain inpatient COVID-19 and COVID-19 related deaths (Supplementary Table 2).

In the UK, the initial COVID-19 testing (i.e., before April 27th 2020) was largely restricted to individuals admitted to hospital care and with typical respiratory infection symptoms, which was later extended to other vulnerable populations and community residents because of increased testing capacity [24]. For example, from July 7th 2020 onward, testing has been routinely offered in all care homes in the UK [25].

#### Covariates

Covariates such as sociodemographic characteristics (e.g., birth year, sex, and race/ethnicity), socioeconomic status (e.g., educational attainment and annual household income), and lifestyle factors (e.g., smoking and alcohol use) were collected at recruitment through touchscreen questionnaires. Body mass index (BMI) was calculated based on the standing height and weight measurement taken at assessment center. Townsend deprivation score, derived based on the postcode of the participant’s registration address, was used to measure material deprivation on a population level, with a greater score representing a greater degree of deprivation [26]. We considered somatic comorbidities as important confounder or mediator for the studied association, and calculated Charlson Comorbidity Index at the time of COVID-19 outbreak for each participant, using the diagnoses of the UK Biobank inpatient hospital data (Supplementary Table 2) [20]. We excluded dementia from this calculation.

### Statistical analysis

The associations between neurodegenerative diseases and different COVID-19 outcomes were assessed by Logistic regression yielding odds ratios (ORs) with 95% confidence intervals (CIs). All models were adjusted for multiple potential confounders, including birth year (as continuous variable), sex (male or female), race/ethnicity (White, Asian, Black, others, or unknown), the Townsend deprivation index (as a continuous variable), BMI (≤24.9 kg/m^2^, 25.0–29.9 kg/m^2^, ≥30.0 kg/m^2^, or unknown), annual household income (<£18,000, £18,000-30,999, £31,000-51,999, £52,000-100,000, >£100,000, or unknown), educational attainment (college degree, A-level, O-level, Certificate of Secondary Education [CSE] or equivalent, National Vocation Qualifications [NVQ] or equivalent, other professional qualifications, or unknown), smoking (never, ever, or unknown), alcohol use (never, previous, current, or unknown), and Charlson Comorbidity Index (as continuous variable). We analyzed first any neurodegenerative disease and then sub-categories of neurodegenerative diseases (primary, vascular, and other neurodegenerative diseases) and specific neurodegenerative diseases (AD and PD).

In subgroup analyses, ORs were calculated by age group (≤66 years, 67–75 years, or ≥ 76 years, according to tertile distribution), sex (male or female), educational attainment (university degree or below), annual household income (<£18,000, £18,000-51,999, or ≥ £52,000), BMI (≤24.9 kg/m^2^, 25.0–29.9 kg/m^2^, or ≥ 30.0 kg/m^2^), smoking (never or ever), alcohol use (never, previous, or current) and Charlson Comorbidity Index (0, 1, or ≥ 2). The difference between sub-group ORs was assessed by introducing interaction terms to the logistic regression or by Wald test.

To explore whether the associations differed by the change of the testing strategy and the dominant variant of the virus in the UK, we did separate analyses for the time periods before April 26th 2020, April 27th 2020 to July 6th 2020, July 7th 2020 to December 16th 2020, and December 17th 2020 onward. Additionally, using the time interval between the first diagnosis of neurodegenerative diseases and cohort entry (< 1 year, 1–2 years, 3–4 years, or ≥ 5 years) as a proxy, we assessed the risk of COVID-19 for individuals at different stages of neurodegenerative diseases. Finally, as a spread of COVID-19 was reported in care homes, mainly from March to early May 2020, in the UK [27], which might have affected more individuals with neurodegenerative disease than individuals without such condition, we further studied the risk of COVID-19 related death among all participants with a positive test result of COVID-19. This analysis was aimed to test whether neurodegenerative disease was associated with the susceptibility to COVID-19, especially the severe form, regardless of chances of being infected or tested.

We also performed a few sensitivity analyses to assess the potential influence of residual confounding and selection bias. Because age is the strongest risk factor for both neurodegenerative diseases and severe COVID-19, any residual confounding due to age might lead to an artificial positive association between neurodegenerative diseases and COVID-19. In a sensitivity analysis, we re-assessed the studied association using a matched cohort design to further control for age. In this analysis, everyone with neurodegenerative diseases was compared with five individuals without neurodegenerative diseases that were individually matched to the exposed individual by birth year and sex and randomly selected from the study population. Further, although we have restricted the main analysis to UK Biobank participants with at least one COVID-19 test, individuals with neurodegenerative diseases might still differ from individuals without such diseases in terms of access to COVID-19 test (e.g., test through inpatient care in the beginning of the pandemic). We therefore performed an additional bias analysis, where we assumed 5–100% underestimate of COVID-19 outcomes among individuals without neurodegenerative diseases and 0% underestimate among individuals with neurodegenerative diseases. Misclassified cases were randomly selected from individuals without neurodegenerative diseases and were repeatedly selected 100 times for each specific percentage (5–100%, by every 5%). We calculated the mean ORs with 95% CIs from these repeated analyses and plotted the distribution of the ORs. All the analyses were conducted by Python software (version 3.8) and R software (version 3.6). A 2-sided *p* < 0.05 was considered statistically significant.

## Results

Among the 96,275 participants included in the analysis, 2617 (2.7%) had a pre-pandemic diagnosis of neurodegenerative disease (exposed group) with the other 93,658 (97.3%) included in the unexposed group (Fig. 1). The mean age at COVID-19 outbreak was 67.9 years and 46.4% of the participants were male (Table 1). We observed little difference in race/ethnicity, BMI, smoking, or alcohol use between the exposed and unexposed groups. However, individuals with neurodegenerative disease were older (74.2 years vs 67.7 years) and more likely to be male (53.0% vs 47.0%), and had lower socioeconomic status (annual household income <£18,000: 34.7% vs 19.8%), lower educational attainment (college or university degree: 20.8% vs 30.0%), greater Townsend deprivation score (− 0.74 vs − 1.20), and higher Charlson Comorbidity Index (2.62 vs 1.29), compared with the unexposed individuals.

**Table 1:** Characteristics of the study cohort

Characteristics	Individuals with neurodegenerative diseases (***n*** = 2617)	Individuals without neurodegenerative diseases (***n*** = 93,658)	Total (***n*** = 96,275)
**Mean age (SD) at outbreak**^a^**, years**	74.2 (5.84)	67.7 (8.23)	67.9 (8.25)
**Age group, years**			
≤ 66	187 (7.1)	30,254 (32.3)	30,441 (31.6)
67–75	436 (16.7)	26,367 (28.2)	26,803 (27.8)
≥ 76	1994 (76.2)	37,037 (39.5)	39,031 (40.5)
**Sex**			
Female	1230 (47.0)	50,369 (53.8)	51,599 (53.6)
Male	1387 (53.0)	43,289 (46.2)	44,676 (46.4)
**Race/ethnicity**			
White	2407 (92.0)	83,989 (89.7)	86,396 (89.7)
Other	189 (7.2)	9136 (9.8)	9325 (9.7)
Unknown	21 (0.8)	533 (0.6)	554 (0.6)
**Body mass index, kg/m**^**2**^			
< 24.9	696 (26.6)	27,367 (29.2)	28,063 (29.1)
24.9–29.9	1082 (41.3)	39,782 (42.5)	40,864 (42.4)
≥ 30	797 (30.5)	25,885 (27.6)	26,682 (27.7)
Unknown	42 (1.6)	624 (0.7)	666 (0.7)
**Smoking**			
Never	1287 (49.2)	48,823 (52.1)	50,110 (52.0)
Ever	1296 (49.5)	44,200 (47.2)	45,496 (47.3)
Unknown	34 (1.3)	635 (0.7)	669 (0.7)
**Alcohol use**			
Never	176 (6.7)	4420 (4.7)	4596 (4.8)
Previous	182 (7.0)	3534 (3.8)	3716 (3.9)
Current	2237 (85.5)	85,364 (91.1)	87,601 (91.0)
Unknown	22 (0.8)	340 (0.4)	362 (0.4)
**Townsend deprivation index**	−0.74 (3.41)	−1.20 (3.12)	−1.19 (3.13)
**Annual household income, £**			
< 18,000	909 (34.7)	18,534 (19.8)	19,443 (20.2)
18,000-30,999	556 (21.2)	19,911 (21.3)	20,467 (21.3)
31,000-51,999	290 (11.1)	20,358 (21.7)	20,648 (21.4)
52,000-100,000	168 (6.4)	15,601 (16.7)	15,769 (16.4)
> 100,000	50 (1.9)	4577 (4.9)	4627 (4.8)
Unknown	644 (24.6)	14,677 (15.7)	15,321 (15.9)
**Educational attainment**			
College or University degree	544 (20.8)	28,109 (30.0)	28,653 (29.8)
A levels/AS levels or equivalent	198 (7.6)	9953 (10.6)	10,151 (10.5)
O levels/GCSEs or equivalent	491 (18.8)	19,969 (21.3)	20,460 (21.3)
CSEs or equivalent	72 (2.8)	5545 (5.9)	5617 (5.8)
NVQ or HND or HNC or equivalent	187 (7.1)	6550 (7.0)	6737 (7.0)
Other professional qualifications	158 (6.0)	4964 (5.3)	5122 (5.3)
Unknown	967 (37.0)	18,568 (19.8)	19,535 (20.3)
**Charlson Comorbidity Index**	2.62 (2.78)	1.29 (2.16)	1.32 (2.19)
**Any COVID-19**			
Yes	718 (27.4)	17,220 (18.4)	17,938 (18.6)
No	1899 (72.6)	76,438 (81.6)	78,337 (81.4)
**Inpatient COVID-19**			
Yes	379 (14.5)	5370 (5.7)	5749 (6.0)
No	2238 (85.5)	88,288 (94.3)	90,526 (94.0)
**COVID-19 related death**			
Yes	195 (7.5)	986 (1.1)	1181 (1.2)
No	2422 (92.5)	92,672 (98.9)	95,094 (98.8)

Data are n/N (%), unless otherwise specified

^a^The first case of COVID-19 was diagnosed on January 31st 2020 in the UK

Any COVID-19 includes a positive test result of COVID-19 according to Public Health England, an inpatient diagnosis from UK Biobank inpatient hospital data, or a death related to COVID-19

**Fig. 2:**
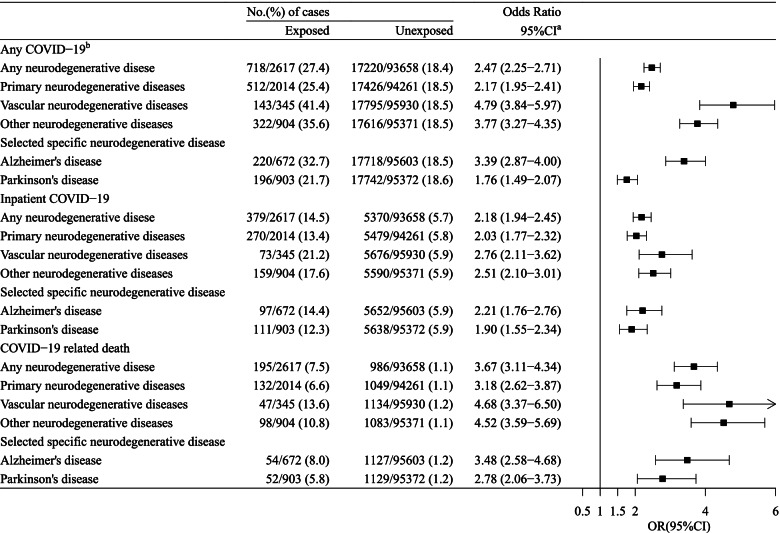
Risk of COVID-19 among individuals with any or specific neurodegenerative disease, compared to individuals without such a condition. ^a^ Analyses were adjusted for birth year, sex, race/ethnicity, body mass index, smoking, alcohol use, Townsend deprivation index, annual household income, educational attainment, and Charlson Comorbidity Index (dementia excluded). ^b^ Any COVID-19 includes a positive test result from Public Health England, an inpatient diagnosis from UK Biobank inpatient hospital data, or a death concerning COVID-19 infection

As of June 14th, 2021, 17,938 individuals were diagnosed with any COVID-19, including 5749 individuals with inpatient COVID-19 and 1181 with COVID-19 related death. We observed an elevated risk of any COVID-19 (27.4% vs 18.4%), inpatient COVID-19 (14.5% vs 5.7%), and COVID-19 related death (7.5% vs 1.1%) among individuals with neurodegenerative disease compared with the unexposed individuals (Table 1). This corresponds to a fully adjusted OR of 2.47 (95%CI 2.25–2.71) for any COVID-19, 2.18 (95%CI 1.94–2.45) for inpatient COVID-19, and 3.67 (95%CI 3.11–4.34) for COVID-19 related death, after adjusting for all available confounders (Fig. 2).

In the analyses for subtypes of neurodegenerative diseases, the elevated risk of any COVID-19 was more pronounced for vascular neurodegenerative diseases, compared with primary neurodegenerative diseases (OR, 4.79 [95%CI 3.84–5.97] vs 2.17 [95%CI 1.95–2.41], *p* value for difference < 0.001) (Fig. 2). No difference was noted for inpatient COVID-19 or COVID-19 related death. We found an increased risk of all studied COVID-19 outcomes among individuals with PD as well as individuals with AD (Fig. 2).

Similar associations were observed across different groups of age, sex, BMI, smoking, alcohol use, annual household income, educational attainment, and Charlson Comorbidity Index (Table 2). A positive association for any COVID-19 was noted during all time periods (OR 1.49 [95%CI 1.13–1.97] before April 26th 2020, OR 4.00 [95%CI 3.11–5.15] during April 27th 2020-July 6th 2020, OR 1.72 [95%CI 1.47–2.01] during July 7th 2020 -December 16th 2020, and OR 2.52 [95%CI 2.15–2.96] from December 17th 2020 onward) (Table 2). No clear trend was observed for time since the first diagnosis of neurodegenerative diseases (Table 3). Finally, among participants with a positive test result for COVID-19, there was also an increased risk of COVID-19 related death among individual with neurodegenerative diseases than others (OR 2.08; 95%CI 1.71–2.53) (Table 4).

**Table 2:** Risk of COVID-19 among individuals with neurodegenerative diseases, compared to individuals without such condition, an analysis of UK Biobank data

Characteristics	No.(%)of any COVID-19 cases		Odds Ratio	No.(%)of Inpatient COVID-19		Odds Ratio	No.(%)of COVID-19 related death		Odds Ratio
	Exposed	Unexposed	(95% CI)	Exposed	Unexposed	(95% CI)	Exposed	Unexposed	(95% CI)
**Sex**									
Female	347/1230 (28.2)	9082/50369 (18.0)	2.81 (2.46–3.21)	164/1230 (13.3)	2519/50369 (5.0)	2.46 (2.06–2.93)	76/1230 (6.2)	355/50369 (0.7)	4.27 (3.26–5.59)
Male	371/1387 (26.7)	8138/43289 (18.8)	2.20 (1.94–2.50)	215/1387 (15.5)	2851/43289 (6.6)	1.99 (1.70–2.33)	119/1387 (8.6)	631/43289 (1.5)	3.37 (2.72–4.17)
**Age, years**									
≤ 66	50/187 (26.7)	8736/30254 (28.9)	1.05 (0.76–1.47)	28/187 (15.0)	1776/30254 (5.9)	2.06 (1.36–3.14)	7/187 (3.7)	87/30254 (0.3)	4.01 (1.63–9.89)
67–75	95/436 (21.8)	4046/26367 (15.3)	1.44 (1.14–1.82)	47/436 (10.8)	1287/26367 (4.9)	1.59 (1.15–2.19)	20/436 (4.6)	219/26367 (0.8)	3.13 (1.91–5.15)
≥ 76	573/1994 (28.7)	4438/37037 (12.0)	2.73 (2.46–3.03)	304/1994 (15.2)	2307/37037 (6.2)	2.25 (1.97–2.57)	168/1994 (8.4)	680/37037 (1.8)	4.03 (3.36–4.83)
**BMI, kg/m**^**2**^									
< 24.9	182/696 (26.1)	4424/27367 (16.2)	3.09 (2.58–3.71)	91/696 (13.1)	1029/27367 (3.8)	3.14 (2.47–3.99)	52/696 (7.5)	130/27367 (0.5)	7.41 (5.21–10.54)
25.0–29.9	317/1082 (29.3)	7203/39782 (18.1)	2.85 (2.48–3.27)	161/1082 (14.9)	2155/39782 (5.4)	2.46 (2.06–2.95)	90/1082 (8.3)	372/39782 (0.9)	4.91 (3.82–6.31)
≥ 30	208/797 (26.1)	5447/25885 (21.0)	1.77 (1.50–2.09)	120/797 (15.1)	2120/25885 (8.2)	1.57 (1.28–1.93)	50/797 (6.3)	462/25885 (1.8)	1.88 (1.37–2.57)
**Smoking**									
Never	325/1287 (25.3)	9076/48823 (18.6)	2.43 (2.12–2.78)	162/1287 (12.6)	2460/48823 (5.0)	2.33 (1.95–2.78)	79/1287 (6.1)	358/48823 (0.7)	4.05 (3.11–5.27)
Ever	381/1296 (29.4)	8033/44200 (18.2)	2.51 (2.21–2.85)	211/1296 (16.3)	2857/44200 (6.5)	2.09 (1.78–2.45)	115/1296 (8.9)	613/44200 (1.4)	3.57 (2.87–4.44)
**Alcohol use**									
Never	48/176 (27.3)	1072/4420 (24.3)	1.75 (1.22–2.51)	30/176 (17.0)	381/4420 (8.6)	1.81 (1.18–2.78)	6/176 (3.4)	79/4420 (1.8)	0.99 (0.41–2.36)
Previous	63/182 (34.6)	693/3534 (19.6)	2.34 (1.68–3.24)	41/182 (22.5)	315/3534 (8.9)	2.13 (1.45–3.14)	20/182 (11.0)	72/3534 (2.0)	3.58 (2.05–6.24)
Current	602/2237 (26.9)	15,385/85364 (18.0)	2.55 (2.31–2.82)	305/2237 (13.6)	4643/85364 (5.4)	2.23 (1.96–2.54)	168/2237 (7.5)	824/85364 (1.0)	4.15 (3.47–4.98)
**Annual household income, £**									
< 18,000	279/909 (30.7)	3501/18534 (18.9)	2.44 (2.10–2.84)	158/909 (17.4)	1490/18534 (8.0)	2.11 (1.75–2.54)	75/909 (8.3)	368/18534 (2.0)	3.05 (2.33–3.99)
18,000-51,999	197/846 (23.3)	7597/40269 (18.9)	2.28 (1.93–2.70)	94/846 (11.1)	2120/40269 (5.3)	2.21 (1.77–2.77)	52/846 (6.1)	335/40269 (0.8)	4.44 (3.25–6.07)
> 52,000	45/218 (20.6)	3560/20178 (17.6)	2.13 (1.52–3.00)	15/218 (6.9)	761/20178 (3.8)	1.84 (1.07–3.16)	15/218 (6.9)	81/20178 (0.4)	8.61 (4.69–15.78)
**Educational attainment**									
University degree	115/544 (21.1)	4307/28109 (15.3)	2.38 (1.92–2.96)	66/544 (12.1)	1170/28109 (4.2)	2.76 (2.10–3.64)	32/544 (5.9)	186/28109 (0.7)	4.70 (3.14–7.04)
Without University degree	290/1106 (26.2)	9310/46981 (19.8)	2.45 (2.13–2.83)	139/1106 (12.6)	2639/46981 (5.6)	2.21 (1.83–2.67)	81/1106 (7.3)	413/46981 (0.9)	4.59 (3.55–5.95)
**Charlson Comorbidity Index**									
0	184/678 (27.1)	10,422/51302 (20.3)	2.59 (2.17–3.09)	80/678 (11.8)	2264/51302 (4.4)	3.17 (2.49–4.05)	46/678 (6.8)	238/51302 (0.5)	8.21 (5.82–11.57)
1	122/485 (25.2)	2760/15181 (18.2)	2.21 (1.78–2.75)	59/485 (12.2)	933/15181 (6.1)	2.22 (1.67–2.96)	31/485 (6.4)	157/15181 (1.0)	4.81 (3.18–7.28)
≥ 2	412/1454 (28.3)	4038/27175 (14.9)	2.33 (2.06–2.64)	240/1454 (16.5)	2173/27175 (8.0)	1.90 (1.64–2.21)	118/1454 (8.1)	591/27175 (2.2)	2.85 (2.30–3.52)
**Calendar period**									
Before April 26th 2020	162/258 (62.8)	1218/2353 (51.8)	1.49 (1.13–1.97)	106/258 (41.1)	1030/2353 (43.8)	0.77 (0.59–1.02)	87/258 (33.7)	295/2353 (12.5)	2.19 (1.61–2.97)
April 27th 2020 to July 6th 2020	101/427 (23.7)	630/7456 (8.4)	4.00 (3.11–5.15)	47/427 (11.0)	325/7456 (4.4)	2.42 (1.73–3.39)	20/427 (4.7)	58/7456 (0.8)	4.02 (2.33–6.93)
July 7th 2020 to Dec 16th 2020	226/1040 (21.7)	7060/35252 (20.0)	1.72 (1.47–2.01)	117/1040 (11.2)	1874/35252 (5.3)	1.85 (1.50–2.27)	41/1040 (3.9)	277/35252 (0.8)	2.46 (1.74–3.49)
Dec 17th 2020 onward	229/892 (25.7)	8312/48597 (17.1)	2.52 (2.15–2.96)	109/892 (12.2)	2141/48597 (4.4)	2.27 (1.83–2.82)	47/892 (5.3)	356/48597 (0.7)	3.42 (2.47–4.75)

Odds Ratio (95%) were derived from logistic regression models, which were adjusted for birth year, sex, race/ethnicity, body mass index, smoking, alcohol use, Townsend deprivation index, annual household income, educational attainment, and Charlson Comorbidity Index

**Table 3:** Risk of COVID-19 among individuals with neurodegenerative diseases, by time since the first diagnosis, compared to individuals without such conditions

	No.(%)of Any COVID-19		Odds Ratio	No.(%)of Inpatient COVID-19		Odds Ratio	No.(%)of COVID-19 related death		Odds Ratio
	Exposed	Unexposed	(95% CI)	Exposed	Unexposed	(95% CI)	Exposed	Unexposed	(95% CI)
**Time since the first diagnosis of neurodegenerative diseases, years**									
< 1	82/309 (26.5)	17,220/93658 (18.4)	2.56 (1.97–3.32)	49/309 (15.9)	5370/93658 (5.7)	2.45 (1.79–3.36)	19/309 (6.1)	986/93658 (1.1)	2.81 (1.73–4.57)
1–2	289/1009 (28.6)		2.70 (2.34–3.12)	158/1009 (15.7)		2.37 (1.98–2.83)	84/1009 (8.3)		3.92 (3.08–5.01)
3–4	145/549 (26.4)		2.42 (1.99–2.95)	72/549 (13.1)		1.95 (1.51–2.51)	49/549 (8.9)		4.45 (3.25–6.09)
≥ 5	202/750 (26.9)		2.27 (1.92–2.68)	100/750 (13.3)		1.99 (1.60–2.47)	43/750 (5.7)		2.89 (2.08–4.00)

Any COVID-19 includes a positive test result from PHE, or an inpatient diagnosis from UK Biobank inpatient hospital data or a death cause of COVID-19 infection

Odds Ratio (95%) were derived from logistic regression models, which were adjusted for birth year, sex, race/ethnicity, body mass index, smoking, alcohol use, Townsend deprivation index, annual household income, educational attainment, and Charlson Comorbidity Index (dementia excluded)

**Table 4:** Individuals with any or specific neurodegenerative disease (exposed), compared to individuals without such a condition (unexposed) - an analysis among all participants with a positive test result for COVID-19

	No.(%) of cases		Odds Ratio^**a**^
	Exposed	Unexposed	
COVID-19 related death			
Any neurodegenerative disease	195/718 (27.2)	986/17220 (5.7)	2.08 (1.71, 2.53)
Primary neurodegenerative diseases	132/512 (25.8)	1049/17426 (6.0)	1.91 (1.52, 2.40)
Vascular neurodegenerative diseases	47/143 (32.9)	1134/17795 (6.4)	1.89 (1.29, 2.77)
Other neurodegenerative diseases	98/322 (30.4)	1083/17616 (6.1)	2.16 (1.65, 2.82)
Selected specific neurodegenerative diseases			
Alzheimer’s disease	54/220 (24.5)	1127/17718 (6.4)	1.58 (1.12, 2.21)
Parkinson’s disease	52/196 (26.5)	1129/17742 (6.4)	1.97 (1.39, 2.78)

^a^ Odds Ratio (95%) were derived from logistic regression models, which were adjusted for birth year, sex, race/ethnicity, body mass index, smoking, alcohol use, Townsend deprivation index, annual household income, educational attainment, and Charlson Comorbidity Index

In the sensitivity analysis using a matched cohort design, including 2617 exposed individuals and 11,571 birth year- and sex- matched unexposed individuals, we obtained largely comparable results (Supplementary Fig. 1) as in the main analysis. The fully adjusted OR was 2.17 (95%CI 1.96–2.41) for any COVID-19, 2.17 (95%CI 1.89–2.48) for inpatient COVID-19, and 3.90 (95%CI 3.17–4.79) for COVID-19 related death. In the bias analysis, we found a positive association between neurodegenerative disease and all COVID-19 outcomes when assuming different degrees of underestimation of COVID-19 outcomes among individuals without neurodegenerative diseases, although the association was not statistically significant for any COVID-19 when assuming > 65% underestimation among individuals without neurodegenerative diseases (Supplementary Fig. 2).

## Discussion

To the best of our knowledge, this is the first cohort study to comprehensively assess the association between multiple neurodegenerative diseases and the risk of COVID-19 related outcomes. Among the UK Biobank participants who received at least one COVID-19 test, we found individuals with a previous neurodegenerative disease to be at an elevated risk of COVID-19, including any COVID-19 infection, hospitalization for COVID-19, and death due to COVID-19. The association was independent from multiple confounders, such as age and somatic comorbidities, and did not differ greatly between primary neurodegenerative diseases and neurodegenerative diseases with a primary vascular component. The subgroup analysis among individuals with a positive test result for COVID-19 showed that, compared to other people, individuals with neurodegenerative disease had further a higher risk of death, after contracting COVID-19. Taken together, these results suggest that neurodegenerative disease is associated with an increased susceptibility to COVID-19, especially the severe form.

The association between neurodegenerative diseases and COVID-19 has been discussued in nine previous studies, with inconsistent findings [14–22]. Four cohort studies, conducted in the UK, Russia, and Italy, reported a positive association between dementia and COVID-19 hospitalization and death [14, 19–21]. Beside a shorter follow-up period and limited number of COVID-19 cases, these studies did not fully control for some important cofounders, such as socioeconomic and lifestyle factors. Similarly, a moderate increase in COVID-19 mortality (30, 95%CI 13–49%) was observed among PD patients in a US cohort study, without however adjusting for comorbidities [22]. Null association was reported in two studies of PD, both with relatively small sample size [16, 17]. Using data from one single hospital in Wuhan, China, patients with AD were found to have a significantly shorter duration of hospital stay for COVID-19 [18]. To this end, the present study, based on a large community-based cohort and with careful consideration of selection bias and a wide range of potential confounders, demonstrated a clear association between various neurodegenerative diseases and a markedly increase risk of COVID-19, especially the severe and fatal form of COVID-19. This highlights the needs of special care or intervention, during the COVID-19 outbreak, for individuals with neurodegenerative diseases.

The underlying mechanisms for the observed association remain unclear. A possible explanation is the generally increased susceptibility to infection, possibly induced by dysregulated immune system inherently involved in the development of neurodegenerative diseases [28–30], including alterations in the peripheral immune responses [31, 32]. In addition, chronic inflammation is commonly observed in neurodegenerative diseases and may also lead to increased risk of infection [30, 31]. We observed slightly higher OR for AD relative to PD, which might imply special pathways linking AD with COVID-19, in addition to common mechanisms for the entire spectrum of neurodegenerative diseases. Based on experimental studies, SARS-CoV-2 binds to the target cell via transmembrane protein angiotensin-converting enzyme 2 (ACE2), which is expressed in various of organs [33]. Given that elevated ACE2 expression level has been observed in AD patients [34], it is possible that the excess in ACE2 in AD provides more cellular receptors when exposed to SARS-CoV-2, and therefore increases the vulnerability to COVID-19.

The major strength of our study is the use of a large community-based cohort, where data on exposure and outcome were collected prospectively and independently. The large sample size made it possible to perform detailed analysis for different subtypes of neurodegenerative disease, as well as for individuals with different characteristics. In addition, the rich information on sociodemographic and medical conditions enabled us to adjust for a wide range of confounders. Also, the application of a matched-cohort design in the sensitivity analysis demonstrated the robustness of our findings to age, as an important confounder. Finally, as multiple data sources were used for the identification of COVID-19 status, we were able to study COVID-19 with different levels of severity, any COVID-19 infection, COVID-19 requiring inpatient care, and COVID-19 related death.

There were several limitations in this study. First, under ascertainment of COVID-19 cases, especially cases with mild or no symptoms, is a concern of all population-based studies for COVID-19. Individuals with chronic diseases (e.g., neurodegenerative diseases) might have different access to health care and therefore different access to COVID-19 test. For example, patients undergo non-COVID-19 related health care might receive additional COVID-19 test. Thus number of potential under ascertainment cases of mild and no symptoms COVID-19 cases in exposed group and unexposed group is likely differential and would lead to selection bias in a study aiming to examine the association between neurodegenerative diseases and COVID-19. As a result, in the main analysis, we restricted the analyses to individuals who had received at least one COVID-19 test, instead of studying the entire UK Biobank population. While the tested population is strongly influenced by the testing policy, especially in the beginning of the pandemic due to the limited testing capacity, they were restricted in the inpatients with typical syndrome of respiratory system. To address this, we further performed a bias analysis, assuming 5 to 100% underestimate of COVID-19 outcomes among individuals without neurodegenerative diseases, whereas no underestimate among individuals with neurodegenerative diseases, and found reassuringly positive associations in even the relatively extreme scenarios. Second, we had no information on place of residence (e.g., care home) for the study participants, which might raise a concern that the noted positive association might have been influenced by the spreading of COVID-19 in care homes, assuming that more individuals with neurodegenerative diseases were residing in care homes than others. However, the outbreak in UK care homes mainly occurred in spring 2020 (i.e., March to early May) [27]. Together, based on data from Whole Care Home Testing Programme, including test results from all 9081 care homes for 65+ in England, the positive rate of COVID-19 tests was as low as 3.9% (6747 out of 172,066) between 11th May and 7th June 2020 [35]. We believe therefore that the spreading of COVID-19 in care homes did not pertain to the entire period of our study. In subgroup analyses by calendar periods, we set the time points according to the changing of test policy (April 27th 2020, July 7th 2020) and the dominant variants in the UK (December 17th 2020) [36]. We observed constantly increased risk of COVID-19 outcomes among individuals with neurodegenerative diseases before April 26th 2020, during April 27th 2020-July 6th 2020, during July 7th 2020 -December 16th 2020, and from December 17th 2020 onward. If the spreading of COVID-19 in care homes indeed contributed heavily to our results, we should have expected a specifically high OR during July 7th 2020 -December 16th 2020, as regular COVID-19 tests have been offered in all care homes in the UK since July 7th 2020 [25]. We believe therefore that the spreading of COVID-19 in care homes was unlikely to explain completely our findings. This concern was further alleviated by the fact that we observed increased risk of COVID-19 related death in relation to neurodegenerative disease in the subgroup analysis of individuals tested positive for COVID-19. Third, somatic comorbidities were measured by diagnoses extracted from inpatient hospital data, as a result we might have missed milder comorbidities (e.g., diabetes) that did not require hospitalization. The adjustment of Charlson Comorbidity Index may not completely relieve the concern on confounding due to differential somatic conditions between individuals with neurodegenerative diseases and the unexposed group. Fourth, with only 5.5% response rate among the target population, the UK Biobank participants are not representative of the general population in the UK. Caution is therefore required when generalizing these findings to the whole UK or other populations [37]. Finally, as we defined neurodegenerative diseases according to inpatient hospital data, our findings might not be directly applicable to individuals with neurodegenerative diseases that are never attended by inpatient care.

## Conclusions

Among the UK Biobank participants who underwent a test for SARS-CoV-2, a pre-existing diagnosis of neurodegenerative diseases was associated with a subsequently elevated risk of COVID-19, especially the severe form. Although validations from future studies are needed, these findings underscore the need for better surveillance and medical care for individuals with neurodegenerative diseases during the COVID-19 outbreak, both before and after contracting the infection.

## Supplementary Information

**Additional file 1 Supplementary Fig. 1**. Risk of COVID-19 among individuals with any or specific neurodegenerative disease, compared to *matched* individuals without such a condition. **Supplementary Fig. 2**. Changes of odds ratios with 95% confidence intervals^a^, by assuming 5–100% underestimation of the studied outcomes among individuals without neurodegenerative diseases. **Supplementary Table 1** A Summary of previous studies addressing the association between neurodegenerative diseases and COVID-19. **Supplementary Table 2**. International Classification of Disease (ICD) codes, ninth (ICD-9) and tenth (ICD-10) revisions for diagnoses used in this study.

## Data Availability

Data from the UK Biobank (http://www.ukbiobank.ac.uk/) are available to all researchers upon making an application. Part of this research was conducted using the UK Biobank Resource under Application 54803.

## Abbreviations

ACE2: Angiotensin-converting enzyme 2
AD: Alzheimer’s disease
BMI: Body mass index
COVID-19: Coronavirus disease 2019
CSE: Certificate of Secondary Education
HES: Hospital Episode Statistics
ICD: International Classification of Diseases codes
NHS: National Health Service
NVQ: National Vocation Qualifications
ORs: Odd ratios
PD: Parkinson’s disease
PEDW: Patient Episode Database for Wales
PHE: Public Health England
SARS-CoV-2: Severe acute respiratory syndrome coronavirus 2
UK: United Kingdom
WHO: World Health Organization
