# Supplementary material for: COVID-19 related outcomes among individuals with neurodegenerative diseases: a cohort analysis in the UK biobank
Source: BMC Neurol. 2022 Jan 7;22:15. doi: 10.1186/s12883-021-02536-7 (PMC8739517; doi:10.1186/s12883-021-02536-7)
Supplement: Supplementary file 1 — Additional file 1 Supplementary Fig. 1. Risk of COVID-19 among individuals with any or specific neurodegenerative disease, compared to matched individuals without such a condition. Supplementary Fig. 2. Changes of odds ratios with 95% confidence intervalsa, by assuming 5–100% underestimation of the studied outcomes among individuals without neurodegenerative diseases. Supplementary Table 1 A Summary of previous studies addressing the association between neurodegenerative diseases and COVID-19. Supplementary Table 2. International Classification of Disease (ICD) codes, ninth (ICD-9) and tenth (ICD-10) revisions for diagnoses used in this study. [file 12883_2021_2536_MOESM1_ESM.docx]

**supplementary material**


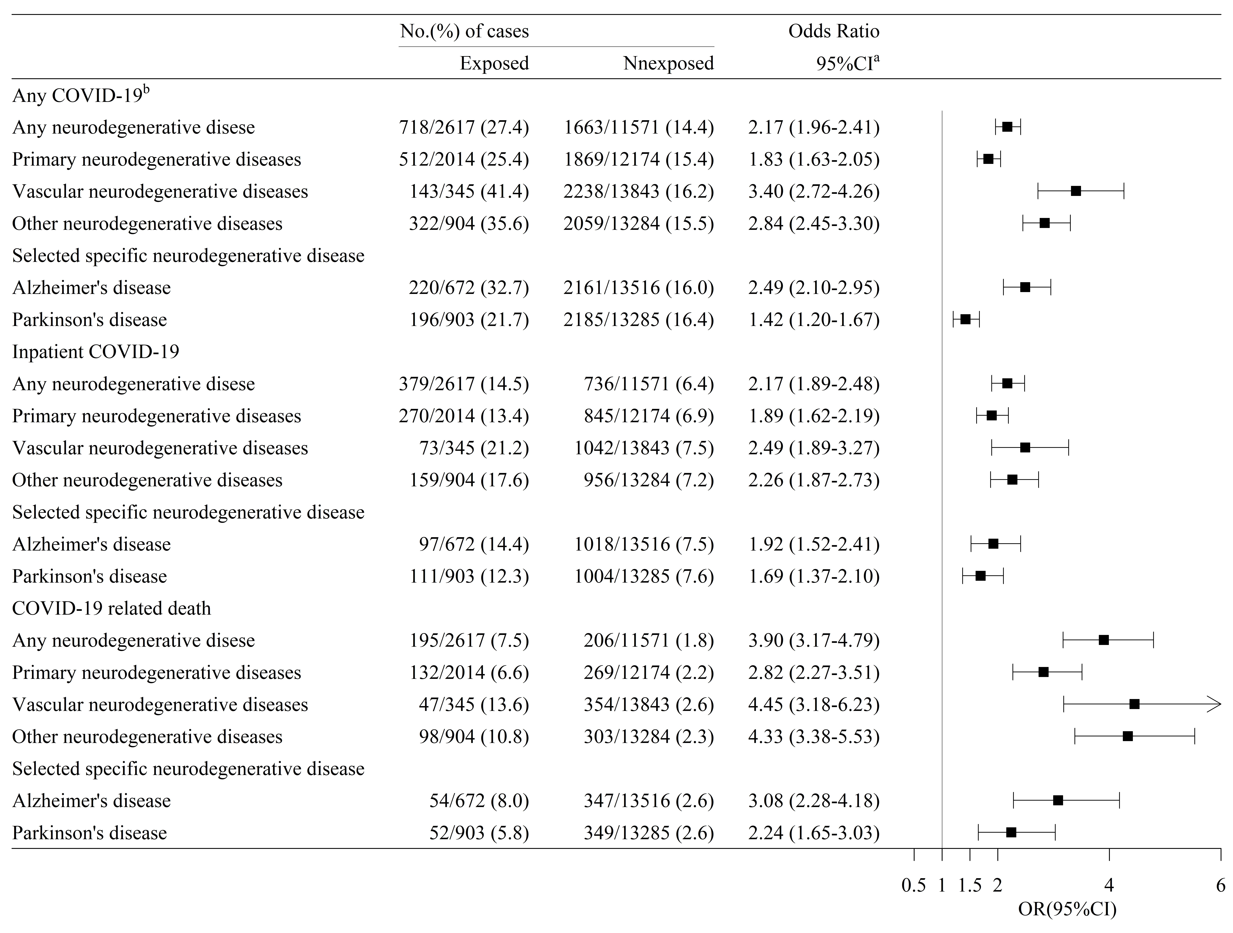


Supplementary Figure 1 Risk of COVID-19 among individuals with any or specific neurodegenerative disease, compared to ***matched*** individuals without such a condition

Odds Ratio (95%) were derived from logistic regression models, which were adjusted for birth year, sex, race/ethnicity, body mass index, smoking, alcohol use, Townsend deprivation index, annual household income, educational attainment, and Charlson Comorbidity Index

Any COVID-19 includes a positive test result from PHE, or an inpatient diagnosis from UK Biobank inpatient hospital data or a death cause of COVID-19 infection.


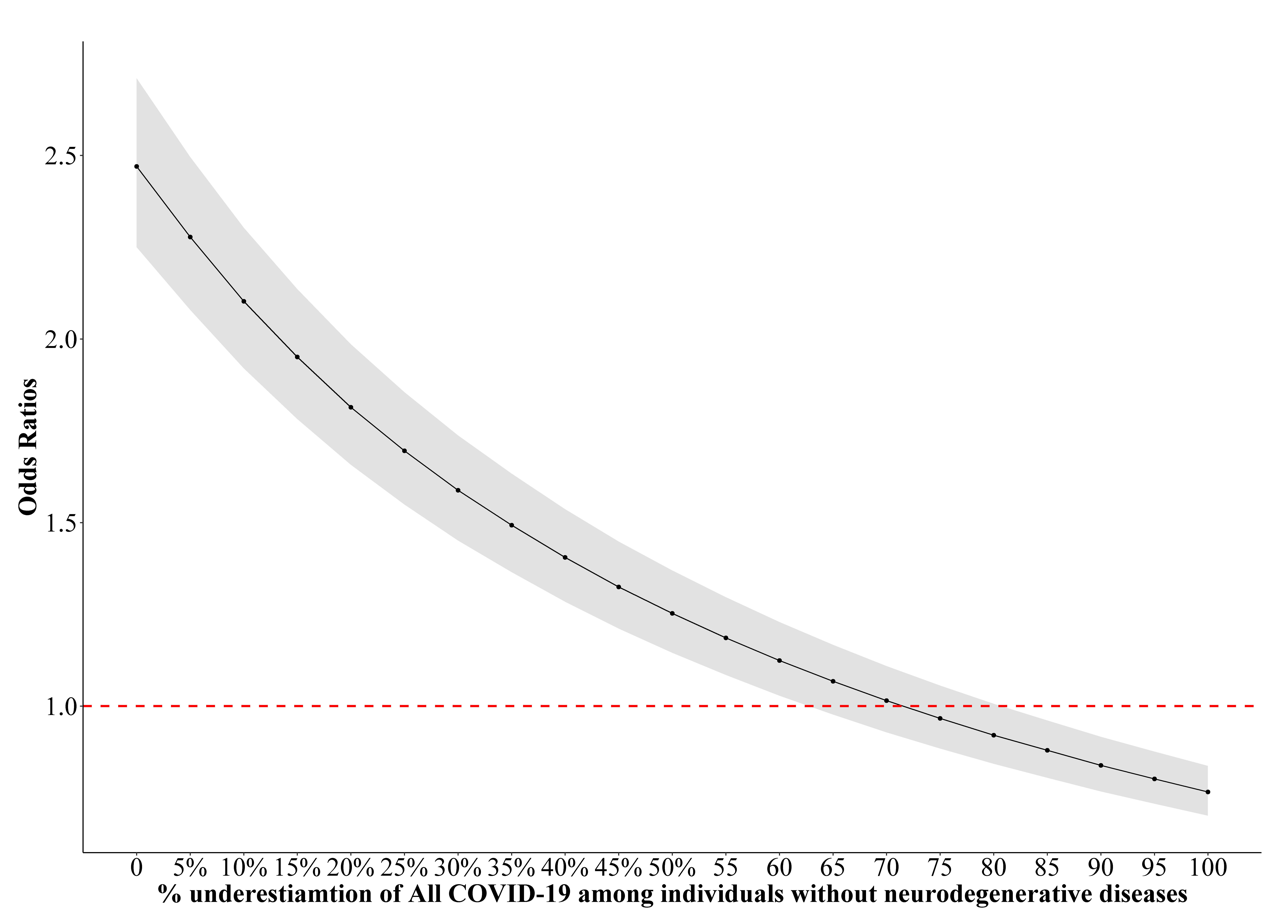


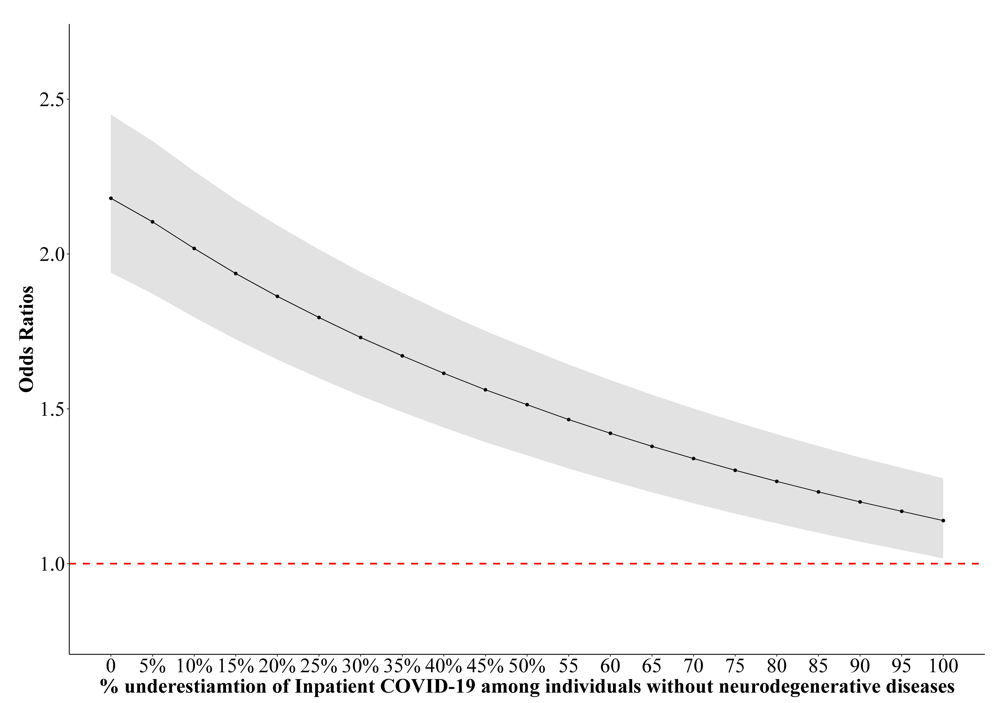


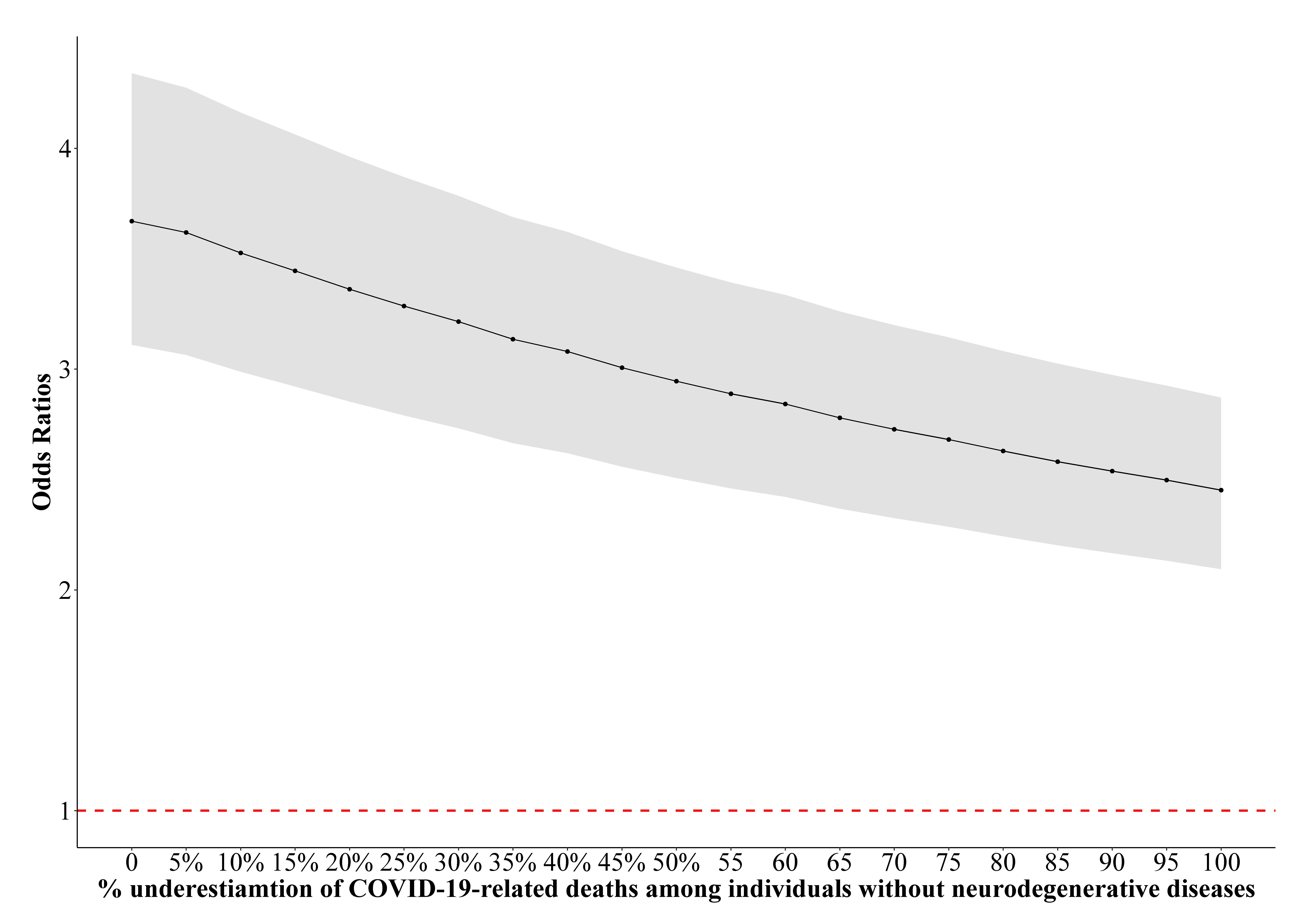


Supplementary Figure 2 Changes of odds ratios with 95% confidence intervals^a^, by assuming 5-100% underestimation of the studied outcomes among individuals without neurodegenerative diseases

Logistic regression models were adjusted for race/ethnicity, body mass index, smoking, alcohol use, Townsend deprivation index, annual household income, educational attainment, and Charlson Comorbidity Index (dementia excluded). Misclassified cases were randomly selected from individuals without neurodegenerative diseases and were repeatedly selected 100 times for each specific percentage (5-100%, by every 5%). We calculated the mean ORs with 95% CIs from these repeated analyses and plotted the distribution of the ORs.

**Supplementary Table 1 A Summary of previous studies addressing the association between neurodegenerative diseases and COVID-19**

| Paper | Study design and data source | Exposure | Outcomes | Sample size | Number of individuals with positive outcome among exposed group | Main findings | Confounders controlled |
| --- | --- | --- | --- | --- | --- | --- | --- |
| Atkins JL 2020^1^ | Prospective cohort study, UK Biobank | Dementia | COVID-19 Positive Inpatient, COVID-19 Positive Inpatient and Death | 269,070 aged over 65 years participants | 7 with positive COVID-19 test | Dementia patients vs. individuals without such condition: OR 3.50 (95%CI 1.93-6.34) for COVID-19 Positive Inpatient, OR 7.30 (95%CI 3.28-16.21) for COVID-19 Positive Inpatient and Death | Age group, sex, ethnicity, education, baseline assessment center, and limited comorbidities |
| Bianchetti A 2020^2^ | Cross-sectional study, COVID-19 hospitalized patients in two Italian hospitals | Dementia | Mortality | 627 hospitalized patients | 51 died among dementia patients | Dementia vs no dementia: 62.2% 26.2%, p<0.001 for mortality | No |
| Fasano A 2020^3^ | Cross-sectional, single  tertiary centre in Lombardy | PD | COVID-19 risk, mortality among COVID-19 patients | 1486 PD patients, 1207 family members | 105 identified as COVID-19, 6 died | PD patients vs family members: 7.1% vs 7.6%, p=0.6 for COVID-19 susceptibility;  PD patients with COVID-19 vs family members with COVID-19: 5.7% vs 7.6% p=0.2 for COVID-19 mortality | Age (only for COVID-19 mortality) |
| Hwang J 2020^4^ | Cross-sectional study, two hospitals in Daegu, South Korea | PD, dementia | COVID-19 mortality | 103 hospitalized patients | 8 of dementia, 1 of PD | COVID-19 death with dementia vs COVID-19 survivor with dementia: 31% vs 4%, p<0.001;  COVID-19 death with PD vs COVID-19 survivor with PD: 4% vs 1%, p=0.416; | No |
| Li J 2020^5^ | Cross-sectional study, a single hospital in Wuhan, China | AD | COVID-19 mortality | 42 COVID-19 inpatients | 2 died | AD patients vs patients without AD:11% vs 13%, p>0.05 for COVID-19 mortality, 9 days vs 24 days, p=0.002 for hospitalization duration | No |
| Giorgi Rossi P 2020^6^ | Prospective cohort study, COVID-19 patients in the Reggio Emilia province, Italy | Dementia | COVID-19 inpatient, mortality | 2,653 patients with all symptomatic COVID-19 | 50 hospitalized, 25 died | COVID-19 patients with dementia vs COVID-19 patients without dementia: HR 1.2 (95%CI 0.9-1.8) for inpatient, HR 1.8 (95%CI 1.1-2.8) for mortality. | Age and sex |
| Dorchety AB 2020^7^ | Prospective cohort study, Public Health  England | Dementia | COVID-19 mortality | 20,133 COVID-19 inpatients | Not provided | COVID-19 inpatients with dementia vs COVID-19 inpatients without dementia: HR 1.40 (95%CI 1.28 to 1.52) for mortality | Age, sex, and limited comorbidities |
| Munblit D 2020^8^ | Prospective cohort study, four adult tertiary university hospitals in Moscow, Russia | Dementia | COVID-19 mortality | 3,480 suspected COVID-19 inpatients | 20 died | Suspected COVID-19 inpatients with dementia vs suspected COVID-19 inpatients without dementia: OR 2.73 (95% 1.34-5.47) for mortality | Age, sex, limited comorbidities |
| Zhang Q 2020^9^ | Retrospective cohort, TriNetX COVID-19 research network | PD | COVID-19 mortality | 79,049 adult COVID-19 patients | 148 died | COVID-19 patients with PD vs COVID-19 patients without PD: OR 1.27 (95% 1.04-1.53) for mortality | Age, sex, race |

COVID-19, coronavirus disease 2019; CI, confidence interval; PD, Parkinson’s disease; AD, Alzheimer’s disease; HR, hazard ratio; OR, odds ratio.

1. Atkins JL, Masoli JAH, Delgado J, et al. Preexisting Comorbidities Predicting COVID-19 and Mortality in the UK Biobank Community Cohort. J Gerontol A Biol Sci Med Sci. 2020;75(11):2224–2230.
2. Bianchetti A, Rozzini R, Guerini F, et al. Clinical Presentation of COVID19 in Dementia Patients. *J Nutr Health Aging*. 2020;24(6):560–562.
3. Fasano A, Cereda E, Barichella M, et al. COVID-19 in Parkinson's Disease Patients Living in Lombardy, Italy. *Mov Disord*. 2020;35(7):1089–1093.
4. Hwang JM, Kim JH, Park JS, Chang MC, Park D. Neurological diseases as mortality predictive factors for patients with COVID-19: a retrospective cohort study. *Neurol Sci*. 2020;41(9):2317–2324.
5. Li J, Long X, Huang H, et al. Resilience of Alzheimer's Disease to COVID-19. *J Alzheimers Dis*. 2020;77(1):67–73.
6. Giorgi Rossi P, Marino M, Formisano D, Venturelli F, Vicentini M, Grilli R. Characteristics and outcomes of a cohort of COVID-19 patients in the Province of Reggio Emilia, Italy. *PloS one*. 2020;15(8):e0238281.
7. Docherty AB, Harrison EM, Green CA, et al. Features of 20 133 UK patients in hospital with covid-19 using the ISARIC WHO Clinical Characterisation Protocol: prospective observational cohort study. *BMJ*. 2020;369:m1985.
8. Munblit D, Nekliudov NA, Bugaeva P, et al. StopCOVID cohort: An observational study of 3,480 patients admitted to the Sechenov University hospital network in Moscow city for suspected COVID-19 infection. *Clin Infect Dis*. 2020:ciaa1535.
9. Zhang Q, Schultz JL, Aldridge GM, Simmering JE, Narayanan NS. Coronavirus Disease 2019 Case Fatality and Parkinson's Disease. *Mov Disord*. 2020;35(11):1914–1915.

Supplementary Table 2. International Classification of Disease (ICD) codes, ninth (ICD-9) and tenth (ICD-10) revisions for diagnoses used in this study

|  |  | **ICD-10** | **ICD-9** |
| --- | --- | --- | --- |
| ***COVID-19*** |  |  |  |
| Inpatient COVID-19 | - | U07.1, U07.2 | - |
| COVID-19 related death |  | U07.1, U07.2 |  |
|  |  |  |  |
| **Neurodegenerative diseases** |  |  |  |
| Any neurodegenerative diseases | Primary neurodegenerative diseases | F00, F02.0, F02.3, G12.2, G20, G23.1, G23.2, G23.8, G23.9, G25.9, G30, G31.0, G31.1, G31.8, G31.9 | 290.0, 290.1, 331.0, 331.1, 331.2, 331.9, 332.0, 333.0, 335.2 |
|  | Vascular neurodegenerative diseases | F01, G21.4 | 290.4 |
|  | Other neurodegenerative diseases | F03, F05.1 | 290.8, 290.9 |
| *Specific neurodegenerative diseases* |  |  |  |
| Alzheimer's disease |  | F00, G30 | 290.0, 290.1, 331.0 |
| Parkinson’s disease |  | G20 | 332.0 |
| **Charlson comorbidity index score** |  |  |  |
| 1 | Myocardial infarction | I21, I22, I25.2 | 410, 412 |
| 1 | Congestive heart failure | I11.0, I13.0, I13.2, I50 | 428 |
| 1 | Peripheral vascular disease | I70, I71, I73.1, I73.8, I73.9, I77.1, I79.0, I79.2, K55.1, K55.8, K55.9, R02, Z95.8, Z95.9 | 440, 441, 443.9, 785.4, V43.4 |
| 1 | Cerebrovascular disease | G45, G46, I60-I69 | 430-438 |
| 1 | Dementia | F00-F03, F05.1, G30, G31.1 | 290 |
| 1 | Chronic pulmonary disease | J40-J47, J60-J67, J68.4, J70, J84.1, J92.0, J96.1, J98.2 | 490-496, 500-505 |
| 1 | Connective tissue disease | M05, M06, M30, M31.5, M32-M34, M35.1, M35.3, M36.0 | 710.0, 710.1, 710.4, 714.0, 714.1, 714.2, 714.8, 714.9, 725 |
| 1 | Ulcer disease | K25-K28 | 531-534 |
| 1 | Mild liver disease | B18, K70.0-K70.3, K70.9, K71.3-K71.5, K71.7, K73, K74, K76.0, K76.2-K76.4, K76.8, K76.9 | 517.2, 517.4-517.6 |
| 1 | Diabetes mellitus | E10.0, E10.1, E10.6, E10.8, E10.9, E10.0, E11.1, E11.6, E11.8, E11.9, E12.0, E121, E12.6, E12.8, E12.9, E13.0, E13.1, E13.6, E13.8, E13.9, E14.0, E14.1, E14.6, E14.8, E14.9 | 250.0-250.2 |
| 2 | Hemiplegia | G04.1, G11.4, G80.1, G80.2, G81, G82, G83.0, G83.1, G83.2, G83.9, G83.3, G83.4 | 342, 244.1 |
| 2 | Moderate/severe renal disease | I12.0, I13.1, N03.2-N03.7, N05.2-N05.7, N18, N19, N25.0, Z94.0, Z99.2 | 582, 583.0-583.2, 583.4, 583.6, 583,7, 585, 586, 588, V420, V56 |
| 2 | Diabetes mellitus with chronic complications | E10.2-E10.5, E10.7, E11.7, E12.7, E13.7, E14.7, E11.2-E11.5, E12.2-E12.5, E13.2-E13.5, E14.2-E14.5 | 250.3-250.5 |
| 2 | Any tumor | C00-C26, C30-C34, C37-C41, C43, C45-C58, C60-C76, C97 | 140-165, 170-175, 179-195 |
| 2 | Leukemia | C91-C95 | 204-208 |
| 2 | Lymphoma | C81-C85, C88, C90, C96 | 200-203 |
| 3 | Moderate/severe liver disease | I85, K70.4, K72, K76.6 | 456.0-456.2, 572.2-572.4, 572.8 |
| 6 | Metastatic solid tumor | C77-C80 | 196-199 |
| 6 | AIDS | B20-B24 | 042-044 |
